# Successful Management of Acquired Hemophilia A Associated with Bullous Pemphigoid: A Case Report and Review of the Literature

**DOI:** 10.1155/2017/2057019

**Published:** 2017-03-28

**Authors:** Quentin Binet, Catherine Lambert, Laurine Sacré, Stéphane Eeckhoudt, Cedric Hermans

**Affiliations:** ^1^Hemostasis and Thrombosis Unit, Division of Hematology, Cliniques Universitaires Saint-Luc, 1200 Brussels, Belgium; ^2^Division of Dermatology, Cliniques Universitaires Saint-Luc, 1200 Brussels, Belgium; ^3^Hemostasis Laboratory, Division of Biological Chemistry, Cliniques Universitaires Saint-Luc, 1200 Brussels, Belgium

## Abstract

*Background*. Acquired hemophilia A (AHA) is a rare condition, due to the spontaneous formation of neutralizing antibodies against endogenous factor VIII. About half the cases are associated with pregnancy, postpartum, autoimmune diseases, malignancies, or adverse drug reactions. Symptoms include severe and unexpected bleeding that may prove life-threatening.* Case Study*. We report a case of AHA associated with bullous pemphigoid (BP), a chronic, autoimmune, subepidermal, blistering skin disease. To our knowledge, this is the 25th documented case of such an association. Following treatment for less than 3 months consisting of methylprednisolone at decreasing dose levels along with four courses of rituximab (monoclonal antibody directed against the CD20 protein), AHA was completely cured and BP well-controlled.* Conclusions*. This report illustrates a rare association of AHA and BP, supporting the possibility of eradicating the inhibitor with a well-conducted short-term treatment.

## 1. Introduction

Acquired hemophilia A (AHA) is a rare condition, with an approximate incidence of 1 case per million per year. It is caused by the spontaneous formation of neutralizing antibodies, mostly immunoglobulins G (IgG), called inhibitors and directed against endogenous factor VIII (FVIII) [1]. The condition is characterized by severe and unexpected bleeding that may prove life-threatening. About half the cases are idiopathic, while the other half appears associated with pregnancy, postpartum, autoimmune diseases, malignancies, or adverse drug reactions [2]. Patients with autoimmune disorders usually exhibit higher inhibitor titers that do not recede spontaneously or following treatment with corticosteroids alone. Further immunosuppressive therapy is thus often needed [3]. We report a case of AHA associated with bullous pemphigoid (BP), a chronic, autoimmune, subepidermal, blistering skin disease. To our knowledge, only 24 documented cases of this association have been reported previously.

## 2. Case Presentation

A 75-year-old man presented himself to the emergency room with an erythematous, warm, swollen, and painful right knee, along with fever and night sweating of recent onset (4 days). He also complained of recurrent subconjunctival hemorrhages and epistaxis and complained of swelling of both wrists that began a month earlier.

The patient was well-known to the hospital's dermatologists since he had presented himself 21 months earlier with tense cutaneous blisters, with a predilection for flexural areas. The diagnosis of BP was then made by compatible histology and direct immunofluorescence, which showed linear IgG and C3 deposition. Serum samples were tested at 1 : 10 dilution on primate esophagus substrate and splitted human skin by means of indirect immunofluorescence. The examination revealed circulating IgG directed against the dermoepidermic junction and taken away by the epidermic side of the junctional dehiscence. To evaluate the disease activity, an ELISA-test was performed, detecting IgG directed against the hemidesmosomal bullous pemphigoid antigens: BP180 (370 RU/mL) and BP230 (322 RU/mL) (positive if ≥20 RU/mL).

The treatment first consisted of methylprednisolone 12 mg daily and topical corticosteroids. Azathioprine (AZA) 50 mg was added one year after diagnosis, as the lesions failed to regress with corticosteroids alone. Before starting AZA, although there was no anamnestic suspicion of an underlying neoplasm, a thoracoabdominal CT-scan was performed to exclude a paraneoplastic origin of the corticoresistant skin lesions. Because the patient developed various undesirable effects, such as biological hepatitis and secondary diabetes mellitus, the following treatment was then implemented: mycophenolate mofetil (MMF) 500 mg daily instead of AZA, decrease in corticosteroid doses, and maximization of topical treatment (diflucortolone valerate 0.3%). As a result, there was clinical improvement with disappearance of cutaneous and mucosal blisters. Hepatic enzymes rapidly normalized and ELISA tests showed near-normalization of anti-BP180 and anti-BP230 titers. Administration of systemic corticosteroids was eventually stopped, with treatment limited to MMF 250 mg daily and topical corticosteroids, without any recurrence of blisters.

Besides BP and a diabetes mellitus secondary to long-term corticotherapy, the patient's medical history was not contributory. Since the patient is an orphan, there was no known family history. His four children were in good health.

The clinical examination was unremarkable except for an inflamed knee locked in flexed position, a painful hematoma of the right thigh, and multiple other hematomas, without any history of trauma. BP was limited to a few small blisters on hands and feet that had appeared recently.

Blood tests revealed inflammation with elevated C-reactive protein at 283 mg/L. Complete blood count was remarkable for a microcytic anemia of WHO Grade II (Hb: 8.6 g/dL) of mixed hemorrhagic and inflammatory etiology. Clotting screening tests revealed an isolated prolongation of the activated partial thromboplastin time (aPTT) at 56.9 sec (local reference range: 25.1–36.5 sec). Failure to correct aPTT by means of a mixing study was indicative of an inhibitor. We then tested and excluded lupus anticoagulant present in plasma and heparin contamination. Further investigations revealed an isolated defect in coagulation FVIII (5%). The inhibitor FVIII titer amounted to 16 Bethesda units (BU)/mL. At that point, a diagnosis of AHA was made. ELISA tests showed a major increase in anti-BP180 (489 RU/mL) and anti-BP230 (399 RU/mL) titers, contrasting with the mild cutaneous symptoms. Articular puncture of the right knee drew 40 mL of dark red blood. An arthroscopic debridement of the joint was performed at a later time point.

**Figure 1:**
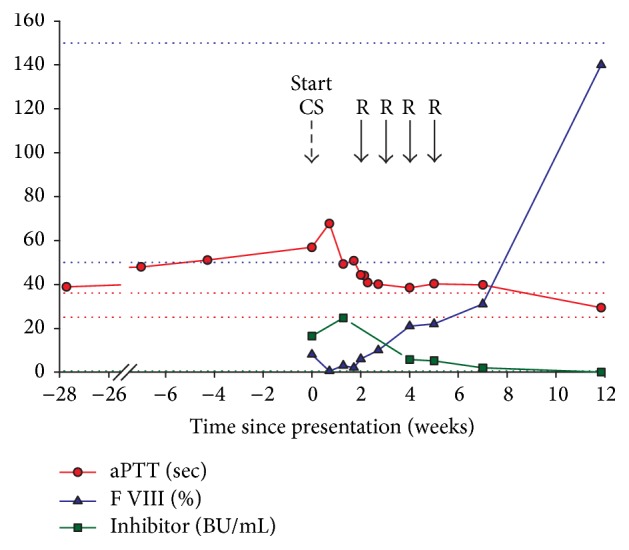
Development of aPTT, factor VIII, and inhibitor titer.* Biological development before and after initial presentation in the emergency room. Dotted lines represent the limit of reference values. Corticosteroids (CS) were administered daily after presentation; rituximab (R) was administered once a week for 4 consecutive weeks (from Day 14)*.

In order to estimate the onset of AHA, we traced back previous clotting tests and found that the aPTT measured 6 months before the onset of AHA was already slightly prolonged (38.9 sec). We therefore assume that the patient developed FVIII inhibitors at least 6 months before bleeding manifestations occurred (Figure 1).

The treatment of AHA consisted in administering methylprednisolone at 1 mg/Kg, which was progressively tapered off over 6 months, together with rituximab 375 mg/m^2^ by intravenous route at weekly intervals for 4 consecutive weeks.

**Figure 2:**
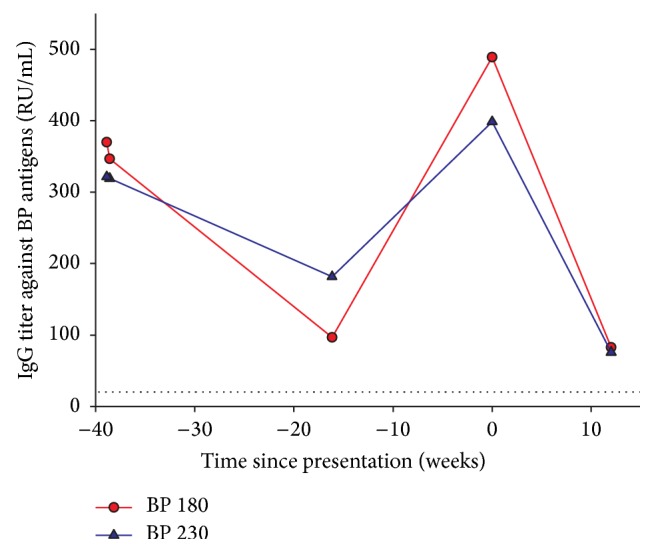
Development of anti-BP180 and BP230 titers.* Follow-up of BP activity before and after AHA. Dotted lines represent the limit of normal values*.

During follow-up, the patient developed oral mucosal bleeding and extensive cheek hematoma, requiring a treatment with tranexamic acid mouthwash and recombinant human coagulation factor VIIa (by-pass therapy with NovoSeven®) at a total dose of 77 mg. Response to treatment was very satisfactory, with inhibitor levels dropping below 6 BU/mL after 4 weeks of treatment and further below 2 BU/mL after 7 weeks. In parallel, plasma FVIII levels improved, without being completely corrected. In less than 12 weeks, we completely eradicated the FVIII inhibitor and restored normal FVIII levels (>100%) and normal aPTT (Figure 1). In the meantime, anti-BP180 and BP230 titers developed favorably as well (Figure 2). Immunosuppressive therapy showed benefits on both AHA and BP, with a complete remission of the conditions. Six months after presentation, the patient was still free from hemorrhagic and cutaneous symptoms.

## 3. Discussion

BP has been reported in association with many skin diseases including psoriasis vulgaris, vitiligo, and squamous cell carcinoma [27, 28]. However, inhibitors of FVIII are an extremely rare complication. The main hypothesis explaining the relationship between BP and AHA is the development of autoantibody cross-reactivity accounted for by a sequence homology between FVIII epitopes and the BP180 collagen XVII domain [16]. In this case report, the concomitant occurrence of sudden bleeding and increased anti-BP titers, in the absence of major cutaneous relapse, supports this statement. We here hypothesize that antibodies directed against BP proteins could cross-react with circulating FVIII, generating AHA along with milder cutaneous symptoms than would be expected with high anti-BP titers. Some authors also suggest that the association between BP and AHA may reflect some underlying immunogenetic susceptibility to autoimmune disease in general [29].

To the best of our knowledge, only 25 documented cases of AHA associated with BP have been reported, including the present one (Table 1). Among these cases, the age distribution ranged from 24 to 88 years of age, with a mean age of 67 years. There was no gender predisposition. BP was usually diagnosed a few months prior to AHA onset, though these two conditions may also develop simultaneously. The mean time between BP and AHA onsets was 6 months, varying from concomitancy to 3 years. None of the AHA cases developed prior to the BP onset. Concomitant improvement and relapse were frequently observed.

The most common symptoms of AHA are extensive bruising, muscle hematomas, and profuse bleeds after trauma or surgery [9]. Our patient, however, consulted the emergency room on account of spontaneous hemarthrosis, which is rarely observed in AHA, unlike standard congenital hemophilia.

The prognosis depends on the severity of hemorrhagic complications and the patient's response to immunosuppression. Poor prognostic factors associated with AHA include old age, comorbidity, and high inhibitor titers (≥20 BU/mL) [30]. The mortality rate of AHA has been estimated at 8–22%, with most hemorrhagic deaths occurring within the first few weeks after presentation [9].

Treatment should be focused on the prevention and treatment of bleeding episodes on the one hand, and on lowering the inhibitor titer on the other. The primary treatment of both AHA and BP is oral corticosteroids. Severe cases may require other immunosuppressive agents like cyclophosphamide and azathioprine [9]. Over the last decade, several small case series have documented successful inhibitor eradication with rituximab, either alone or in combination with standard treatment [31]. However, approximately 20% of patients will likely experience a relapse within 1 week to 14 months after immunosuppressive therapy discontinuation [32]. Long-term follow-up is thus mandatory in AHA patients.

This review also reminds us that the treatment of AHA and BP may require high doses of immunosuppressive drugs, with a risk of significant undesirable effects, such as infection, sepsis, and neutropenia [3].

In conclusion, AHA should be suspected when a patient with no previous personal or family history of bleeding presents himself with bleeding and an isolated aPTT prolongation, especially if he is suffering from an autoimmune disease. The primary treatment of AHA consists in administering oral methylprednisolone. Only three of the 25 patients described in the literature, however, displayed a good response to corticosteroids given alone. Other immunosuppressive drugs should thus be also considered, in particular weekly intravenous injections of rituximab. The two main goals are (1) to treat and prevent bleeding complications and (2) to eradicate the inhibitor [33]. Long-term follow-up proves essential, even after complete inhibitor eradication.

## Figures and Tables

**Table 1:** Reported cases of acquired hemophilia A associated with bullous pemphigoid in the literature.

Number [Ref.]	Age	Sex	Onset BP	Evolution of BP under treatment	Max. inhib. titre (BU/mL)	Treatment of AHA	Evolution of AHA under treatment
1 [4]	74	M	Concurrently with AHA	Good	110	CS, CsA, AZA, CPA, BA, IVIg, FVIII	Clinical and biological remission
2 [5]	68	M	6 months before AHA	Rapid response to topical CS	>2	CS	Clinical and biological remission without recurrence over 12 months
3 [6]	47	F	3 months before AHA	Stable remission	2.04	CS, CPA, PP	Life-threatening complications followed by stable remission
4 [7]	88	M	Few days before AHA	Improved with systemic and topical CS, doxycycline, nicotinamide	(+)	CS, BA	Died shortly after diagnosis
5 [8]	65	M	2-3 months before AHA	AHA occurred at BP relapse	2	CS	Good
6 [8]	67	F	6 months before AHA	Relapsed after self-discontinuation	76	CS, CS pulse, CPA, FFP, FVIII	Good
7 [9]	78	M	4 months before AHA	Resolved with CS	839	CS, CPA, BA	Relapse 3 months after withdrawing of CPA because of severe neutropenia Remission obtained with CS alone for 12 months
8 [10]	71	F	ND	ND	(+)	CS	Died of pulmonary embolism
9 [11]	49	F	7 months before AHA	Resolved with CS, CPA	148	CS, CPA, FFP, PE	Good
10 [12]	71	M	Concurrently with AHA	Resolved with CS	219	CS, IVIg, cryoprecipitate, BA	ND; patient transferred to another hospital.
11 [13]	83	F	3 years before AHA	Controlled with topical CS but relapsed	17	CS, BA	Died of severe hemorrhage
12 [14]	84	F	2 months before AHA	ND	29	CS, CPA, BA	Good, but died of sepsis.
13 [15]	81	F	4 weeks before AHA	Slight improvement with topical CS	7	/	Good, but died of ischemic heart disease
14 [16]	68	F	Concurrently with AHA	Resolved with topical CS	1.4	BA	Good
15 [17]	38	F	Before.	ND	2.44	CS, BA	ND.
16 [18]	64	M	4 weeks before AHA	Improved with systemic and topical CS, doxycycline, nicotinamide	(+)	CS, rituximab, BA	Remission; relapse after a few months, multiple transfusions, died of myocardial infarction
17 [19]	24	M	2 years before AHA	Improved with CS	256	CS, CS pulse, CPA, PP, rituximab, BA	Improved after 2 months
18 [20]	72	M	9 months before AHA	Resolved with MTX and topical CS	200	CS, rituximab, BA	Complete remission
19 [21]	60	F	Concurrently with AHA	Resolved	(+)	CS, CPA, FFP, BA, IVIg	Complete remission
20 [22]	88	M	4 months before AHA	Not improved with CS	7	CS, rituximab, FFP	Remission of BP and AHA, but died of severe pneumonia
21 [23]	49	F	4 months before AHA	Minimal response to CS and IVIg	17	CS, CPA, BA, FVIII	Complete remission
22 [24]	80	F	12 months before AHA	Resolved with CS before AH	20	CS	Biological remission, even after CS discontinuation
23 [25]	73	M	Concurrently with AHA	Good	(+)	CS, CPA, Rituximab, IVIg	Complete remission
24 [26]	61	M	1 month before AHA	Good	32	CS, BA	Clinical and biological improvement
25 [*∗*]	75	M	21 months before AHA	Controlled with systemic and topical CS + AZA/MMF	25	CS, Rituximab, BA	Complete remission

The cases are presented in order of publication date. ND: not described; gender: M(ale)/F(emale); CS: corticosteroid; CsA: ciclosporin; AZA: azathioprine; CPA: cyclophosphamide; FFP: fresh frozen plasma; PE: plasma exchange; PP: plasmapheresis; BA: bypassing agents, for example, FEIBA (Factor Eight Inhibitor Bypassing Activity) or rFVII (recombinant Factor Seven); MTX: methotrexate; *∗*: our case report.
